# Metastatic renal cell carcinoma: risk of rapid progression to pathological fractures and factors influencing post-treatment survival – a Swedish Nationwide cohort study

**DOI:** 10.1016/j.jbo.2026.100749

**Published:** 2026-02-12

**Authors:** Josefin Åkerstedt, Tova Åström, David Wennergren, Johan Wänman

**Affiliations:** a Department of Diagnostics and Intervention, Orthopaedics, Umeå University Hospital, Umeå, Sweden; b Department of Orthopaedics, Spine Surgery, Umeå University Hospital, Umeå, Sweden; c Department of Orthopaedics, Institute of Clinical Sciences, Sahlgrenska Academy, University of Gothenburg, Sweden

**Keywords:** Renal cell carcinoma, Pathologic fractures, Swedish fracture registry

## Abstract

**Background:**

Renal Cell Carcinoma (RCC) represents about 2% of all cancers in Sweden. While RCC is known for its potential to metastasize to the skeleton, few studies have examined the risk factors for pathological fractures and their impact on survival. The Fuhrman grade and AJCC staging systems are commonly used to assess disease aggressiveness, but their relationship to fracture risk and post-fracture outcomes remains unclear.

**Methods:**

This study cross-linked data from the Swedish Fracture Register (SFR) and the Swedish Renal Cancer Register (SNR) to identify RCC patients with pathological fractures. Fracture characteristics, cancer staging (Fuhrman, TNM, AJCC), and histological subtypes were analysed. Survival was estimated using the Kaplan-Meier method.

**Results:**

One hundred four RCC patients (72 men, 32 women) with pathological fractures were included, with clear cell carcinoma being most common (n = 93). Fuhrman grade 3 (n = 34) and fractures in the femur (n = 44) and humerus (n = 37) predominated. Median time from cancer diagnosis to fracture was 11 months. Higher Fuhrman grade, TNM, and AJCC stages were significantly associated with shorter time to fracture (p < 0.001). The median overall survival was 31 months and was related to Fuhrman grade (p = 0.013) and AJCC stage (p < 0.001). The median survival post-fracture was 8 months, with no significant association with cancer grade or stage.

**Conclusion:**

Advanced Fuhrman grade and AJCC stage predict faster progression to pathological fractures in RCC. Given the poor prognosis after pathological fractures, early risk stratification and individualized treatment approaches are essential to improve outcomes.

## Highlights

• Pathological fractures in RCC signal poor prognosis, with a median post-fracture survival of 8 months.
• Higher Fuhrman grade and AJCC stage are linked to quicker onset of pathological fractures, indicating more aggressive disease progression.
• Femur and humerus were the most frequent fracture sites, highlighting common skeletal targets of RCC metastases.

## 1 Introduction

Globally, renal cell carcinoma (RCC) ranks as the fourteenth most common cancer, predominantly affecting men with a peak incidence between 60 and 70 years of age [1]. In Sweden, RCC accounts for approximately 2% of all cancers [2]. The three main types of RCC are clear cell (ccRCC), papillary (pRCC), and chromophobe (chRCC). Some studies have found different affinities for developing skeletal metastases among these groups [3]. Approximately 20–35% of all patients with metastatic renal cell carcinoma (mRCC) exhibit bone involvement [4]. Skeletal metastases arising from RCC were shown to be highly destructive, primarily due to osteolytic processes. These processes increase the risk of skeletal instability, which makes the bones susceptible to pathological fractures that often require surgical intervention [5]. The orthopaedic management of metastatic bone disease primarily aims to relieve pain and enhance mobility and function [6], [7]. RCC exhibits a unique oncological profile, where patients presenting with solitary metastatic lesions, particularly to bone, can achieve prolonged survival following radical surgical resection. This encourages tumour orthopaedic surgeons to frequently manage these isolated metastases similarly to primary tumour eradication [8].

The surgical approach must be durable in relation to the patient’s life expectancy. The intent is to minimize prolonged hospital stays or extended rehabilitation periods, even when fracture healing is uncertain [9], and to reduce the risk for reoperation. Surgical treatment is associated with a risk of complications, morbidity, and mortality, which must be balanced against the potential benefit for the patient [10], [11].

Few studies have investigated the risks associated with the rapid development of pathological fractures in RCC patients or in the survival outcomes following treatment [12]. It was suggested that the grading of RCC, as defined by the Furman or American Joint Committee on Cancer (AJCC) staging, is associated with a more aggressive disease course [13], [14]. However, the relationship between these factors and rapid progression of skeletal metastases or survival outcomes remains unclear.

The objectives of the study were to determine the variables associated with rapid progression from diagnosis to pathological fracture, the anatomical distribution of these fractures, and the factors that influence survival outcomes following treatment in patients with pathological fractures secondary to RCC.

## 2 Materials and methods

**Fig. 1:**
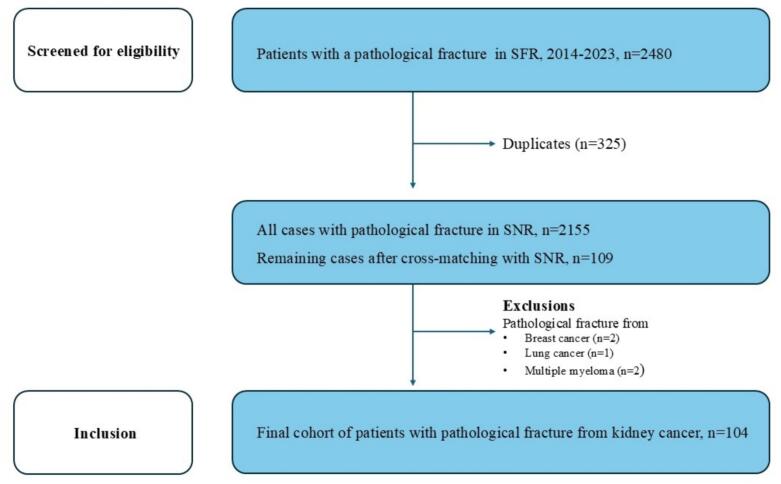
Flowchart illustrating patient selection.

The Swedish Fracture Registry (SFR) is a national, population-based registry that records fractures of all types. Since its establishment in 2011, the registry has collected data on over one million fractures, which provides an excellent foundation for large-scale, registry-based studies. Pathological fractures have been included in the registry since 2006. The SFR currently reports 100% coverage and up to 85% completeness for specific fracture categories [15]. The registration of pathological fractures (International Classification of Diseases, 10th Revision, ICD-10 M84.4.A-G) in the SFR includes the specification of the primary tumour. In the current study, only those fractures registered as kidney cancer were included (Fig. 1).

The Swedish National Quality Register for Renal Cell Carcinoma (SNR) was established in 2005. Unlike the Swedish National Cancer Register, where reporting is mandated by law, the SNR is a voluntary national quality register that specifically collects detailed data on RCC such as the tumour, node, and metastasis (TNM) stage, morphology, malignancy grade, and treatment. In 2022, SNR had a coverage rate of 98% [16].

From the SFR data on injury type and date, age at the time of fracture, anatomical location of the pathological fracture, fracture classification, and type of treatment were extracted. Further, detailed information about date of primary diagnosis, Fuhrman stage, TNM-classification, histological subtype, and age at primary diagnosis were retrieved.

### 2.1 Patient selection

Patients registered in the SFR with a pathological fracture due to metastasis from RCC between January 1, 2014, and December 31, 2023, were identified. To confirm the diagnosis of RCC, the data were cross-matched with the SNR. In cases of duplicate registrations in SFR, all surgeries performed after the primary procedure were excluded.

### 2.2 The AJCC staging system

The American Joint Committee on Cancer (AJCC) staging system is the most commonly used for RCC, with the latest version released in 2018 [17]. The system is based on TNM staging and is divided into stages I-VI based on tumour size, invasiveness of growth pattern, and metastasis [17].

### 2.3 The Fuhrman grading

The Fuhrman nuclear grading system is a widely used histological grading for RCC based on nuclear size, shape, and nucleolar prominence. Tumours are graded from 1 to 4, with higher grades indicating larger more irregular nuclei and more prominent nucleoli. This grading correlates closely with tumour aggressiveness and patient prognosis [18].

### 2.4 Statistics

Categorical variables were presented as frequencies, and a Chi-squared test was performed to compare groups. Continuous variables were presented as medians and/or means with standard deviations (SD). The Kaplan-Meier estimator was used to estimate survival after surgery, overall survival, and time between primary diagnosis and pathological fracture. The log-rank test was applied to test differences in these. Cox proportional hazard regression was used to perform a multivariate analysis and was presented with adjusted hazard ratios and a 95% confidence interval (CI). A p-value of < 0.05 was considered statistically significant. All analyses were performed using IBM SPSS Statistics, version 29.0.2.0 [19].

## 3 Results

### 3.1 Clinical characteristics at the time of primary diagnosis of kidney cancer

The final cohort consisted of 104 patients (72 males and 32 females). ccRCC was the most common type of kidney cancer (n = 93), with 57 tumours located in the left kidney and 47 tumours located in the right kidney. The mean age at the time of primary diagnosis of kidney cancer was 67 years (12 SD). The mean size of the primary tumour was 85 mm (34 SD), with the smallest tumour measuring 20 mm and the largest measuring 170 mm. Fuhrman grade 3 was the most common grade (n = 34), followed by Fuhrman grade 2 (n = 14). Primary surgery for kidney cancer was performed in 52 patients, while oncological treatment was administered to 38 patients at the time of diagnosis (Table 1).

**Table 1:** Clinical characteristics of the study cohort (n = 104) at the time of primary diagnosis of kidney cancer.

**Location**	
Right	47
Left	57
Bilateral	0

**Type of kidney cancer**	
Clear cell carcinoma, ccRCC	93
Chromophobe, chRCC	1
Papillary, pRCC	1
Other	2
Missing	7

**TNM**^a^**classification**	
T1a < 4 cm located in the Kidney	7
T1b 4–7 cm located in the kidney	19
T2 > 7 cm located in the kidney	2
T2a > 7 < 10 cm located in the kidney	8
T2b > 10 cm located in the kidney	10
T3a Tumour thrombosis locally invasive in the kidney	33
T3b Tumour thrombosis in v. cava inferior to the diaphragm	8
T3c Tumour thrombosis in v. cava above the diaphragm	2
T4 Tumour growth extending to Gerota’s fascia	3
TX Not sufficient material for classification	12
N0 No lymph node metastases	71
N1 Single lymph node metastases	22
N2 More than 1 lymph node metastases	9
Nx lymph node was not examined	2
M0	30
M1	71
MX was not examined at the time of primary diagnosis	3

**Fuhrman grading**	
Grade 1	1
Grade 2	14
Grade 3	34
Grade 4	6
Grade X/Missing	28/21

**AJCC**	
Stage 1	13
Stage 2	5
Stage 3	16
Stage 4	70

**Primary treatment of kidney cancer**	
Dead before treatment	3
Expectancy	2
No treatment or palliation	2
Surgery/Ablation	52
Oncological treatment	38
Missing	7

a TNM- Tumor, Node, Metastasis −classification at the time of primary diagnosis.

### 3.2 Pathological fractures

**Fig. 2:**
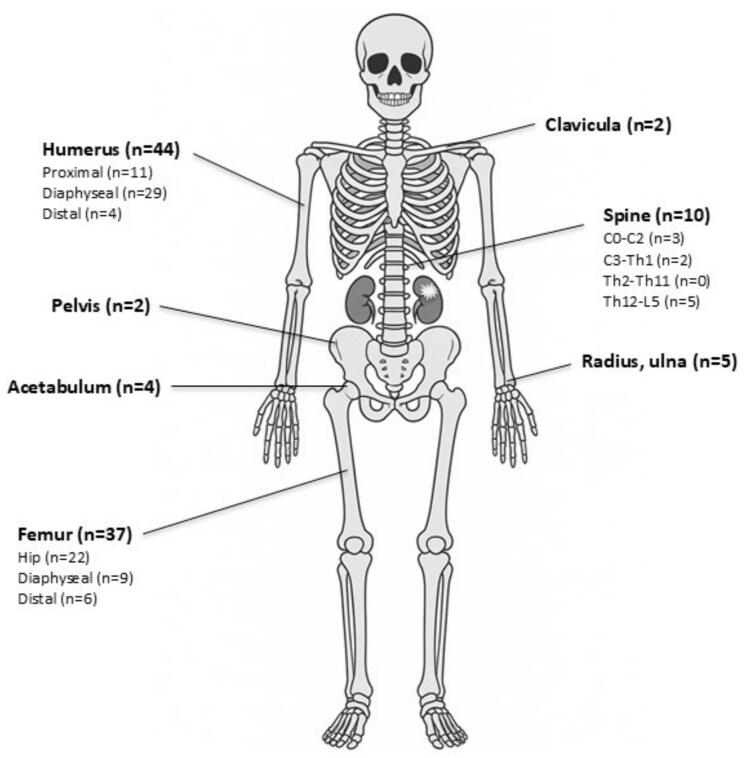
Schematic illustration of the most common sites for pathological fractures.

The mean age at the time of the pathological fracture was 70 years (10 SD). The majority of the pathological fractures occurred in the humerus (n = 44), followed by the femur (n = 37) (Fig. 2). Fuhrman grades were not associated with the location of the pathological fractures (p = 0.17).

Non-surgical treatment was applied to 18 fractures (2 pelvic, 3 humeral diaphysis, 2 distal humerus, 2 clavicle, 4 radius/ulna, and 5 in the spine). Four fractures of the humeral diaphysis underwent surgical intervention following failure of non-surgical treatment. In total, 69 patients received surgical treatment that included all fractures in the femur diaphysis (n = 9) and distal femur (n = 6), as well as 18 out of 22 hip fractures. All patients with fractures of the proximal humerus (n = 11) underwent surgery, as well as 20 out of 29 patients with fractures of the humeral diaphysis. Table 2 summarises the clinical characteristics of the studied cohort at the time of pathological fracture.

**Table 2:** Clinical characteristics of the study cohort (n = 104) at the time of pathological fracture.

Male	72
Female	32

**Location**	
Acetabulum	4
Pelvis	2
Femur (Hip/Diaphysis/Distal)	22/9/6
Humerus (Proximal/Diaphysis/Distal)	11/29/4
Clavicula	2
Spine (C0-C2; C3-Th1; Th2-Th11; th11-L5)	3; 2; 0; 5
Radius/ulna	5

**Fracture treatment**	
Non-surgical treatment	18
Surgical after failed non-surgical treatment	2
Primary surgery	67

**Type of treatment**	
Non-surgical treatment	18
Intramedullar nail	40
Plate fixation	11
Prothesis	16
Spine/Posterior decompression with fusion	2
Spine/Anterior decompression	1

### 3.3 Variables associated with rapid progression towards pathological fractures

**Fig. 3:**
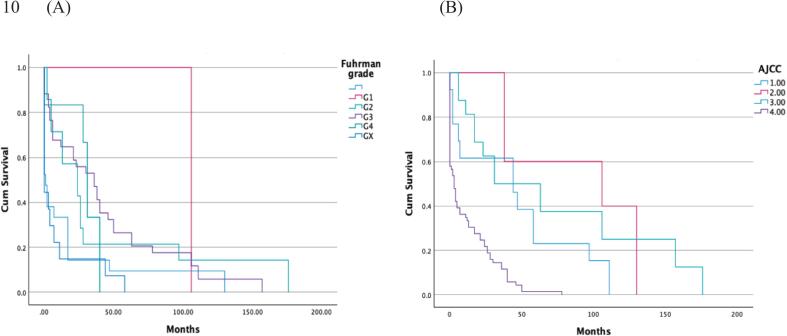
Time from primary diagnosis to pathological fracture. The progression pace towards a pathological fracture from primary diagnosis was associated with both Fuhrman grade (A) (p = 0.001), and AJCC stage (B) (p < 0.001).

The overall median time from diagnosis to pathological fracture was 11 months (95% CI 3–19). Fractures of the radius and ulna had the longest median duration of 78 months (95% CI 0–239), followed by spinal fractures at 28 months (95% CI 13–42) and femoral fractures at 11 months (95% CI 0.7–21); humerus fractures had a median duration of 6 months (95% CI 0.3–12) and pathological pelvic fractures a median duration of 2 months (95% CI 0–4) (p = 0.24). The time of developing a pathological fracture from primary diagnosis was significantly associated with the Fuhrman grade (p = 0.001) (Fig. 3). A higher grade in TNM- classification for all components T (p < 0.001), N (p < 0.001), and M (p < 0.001) at the time of primary diagnosis were associated with a more rapid course towards a pathological fracture. The AJCC was also associated with a rapid course from diagnosis to pathological fracture with a median duration of 3 months (95%CI 0.6–5.4) for grade 4, 31 months (95% CI 0–70) for grade 3, 106 months (95% CI 0–252) for grade 2, and 44 months (95% CI 0–91) for grade 1 (p < 0.001) (Fig. 3). Both high Fuhrman grade and AJCC stage were associated with a rapid progression to pathological fracture in the cox proportional hazard model (Table 3).

**Table 3:** Variables (AJCC stage, Fuhrman grade, sex and age) and their association with rapid progression towards pathological fracture.

**Variable**	**Hazard Ratio (HR)**	**95% Confidence Interval (CI)**	**P-value**
**AJCC Stage**			**<0.001**
Stage I (Ref)	Ref	Ref	Ref
Stage II	0.252	0.067 – 0.951	0.042
Stage III	0.972	0.403 – 2.345	0.949
Stage IV	5.121	2.470 – 10.618	<0.001

**Fuhrman Grade**			**<0.001**
Grade 1 (Ref)	Ref	Ref	Ref
Grade 2	0.213	0.095 – 0.479	<0.001
Grade 3	0.229	0.115 – 0.456	<0.001
Grade 4	0.238	0.082 – 0.688	0.008
Grade X	0.854	0.454 – 1.606	0.623

**Sex (male vs. female)**	0.911	0.544 – 1.525	0.724
**Age at Diagnosis**	0.989	0.972 – 1.007	0.232

### 3.4 Survival

**Fig. 4:**
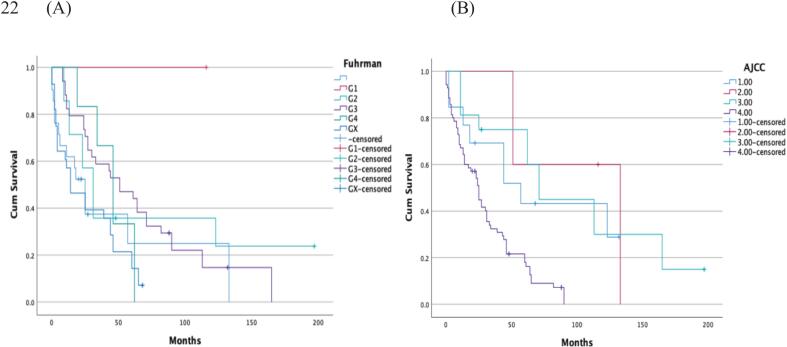
Survival after pathological fracture. The Fuhrman grades (A) (p = 0.027) and AJCC stage (B) (p < 0.001) were significantly associated with survival after primary kidney cancer diagnosis.

The median survival after primary diagnosis of RCC was 31 months (95% CI 14–48 months). In the Kaplan-Meier survival analysis, both Fuhrman grade (p = 0.027) and AJCC stage (p < 0.001) were significantly associated with survival following primary RCC diagnosis (Fig. 4). However, in the multiple Cox proportional hazard model only AJCC (p < 0.001) remained independently associated with survival (Table 4).

**Table 4:** Variables (AJCC stage, Fuhrman grade, sex and age) and their association with survival after primary diagnosis.

**Variable**	**Hazard Ratio (HR)**	**95% Confidence Interval (CI)**	**P-value**
**AJCC Stage**			**<0.001**
Stage I (Ref)	Ref	Ref	Ref
Stage II	0.514	0.126 – 2.087	0.352
Stage III	0.891	0.334 – 2.377	0.818
Stage IV	3.252	1.466 – 7.215	0.004

**Fuhrman Grade**			0.083
Grade 1 (Ref)	Ref	Ref	Ref
Grade 2	0.375	0.152 – 0.928	0.034
Grade 3	0.367	0.173 – 0.780	0.009
Grade 4	0.539	0.189 – 1.532	0.246
Grade X	0.710	0.332 – 1.519	0.378

**Sex (**male vs. female**)**	0.799	0.466 – 1.372	0.416
**Age at Diagnosis**	0.992	0.975 – 1.010	0.397

### 3.5 Survival after treatment of pathological fracture

The median survival after a pathological fracture from kidney cancer was 8 months (95% CI 5–11). There was no significant association between non-surgical or surgical treatment and survival after pathological fracture (p = 0.89). Also, anatomical location of the pathological fracture (p = 0.57), Fuhrman grade (p = 0.85), and AJCC stage (p = 0.89) were not significantly associated with survival after pathological fracture.

## 4 Discussion

This national registry-based study provides insight into the skeletal metastatic behaviour and survival outcomes of patients with RCC who sustained pathological fractures. Importantly, this study provides novel insight into the timing of pathological fracture development in RCC, a specific endpoint that has not been previously characterized in large, population-based cohorts. Our findings indicate that pathological fractures most commonly involve the humerus and femur, and ccRCC was the predominant histological subtype. Tumour grade (Fuhrman) and stage (AJCC) were significant predictors of time to pathological fracture, highlighting a temporal dimension of skeletal disease progression that further underscores their potential utility in risk stratification of RCC patients.

The short median survival of eight months following a pathological fracture highlights the advanced disease state associated with pathological fracture in RCC patients. This aligns with prior research findings of poor prognosis associated with skeletal-related events (SREs) in RCC patients [3]. Whereas the overall prognosis of RCC remains unfavourable, few studies have evaluated predictors for the development of pathological fractures**.** Most existing literature has focused on skeletal-related events or the presence of bone metastases as composite or binary outcomes, rather than on pathological fractures as a distinct endpoint or on the timing of fracture development. Chandrasekar et al. (2017) identified advanced TNM stage and high Fuhrman grade as significant predictors of metastases at diagnosis in patients with RCC [20]. Building on these findings, the present study addresses this gap by examining factors associated with rapid progression from primary RCC diagnosis to pathological fracture in a national population-based cohort. Our findings extend these observations by emphasizing their association with accelerated progression from primary diagnosis to pathological fracture.

Bone metastases in RCC patients are typically lytic and associated with high rates of SREs. Prior studies have shown that targeted interventions can reduce SRE incidence in RCC patients with bone metastases. For example, Bongiovanni et al. (2018) evaluated the combination of denosumab, a RANKL inhibitor, with anti-angiogenic therapies in patients with mRCC. The authors found that denosumab effectively prevented SREs in this patient population, which demonstrates its potential as a targeted intervention to reduce skeletal complications in RCC patients with bone metastasis [21]. Recognizing that risk is closely connected to biological features like tumour grade and histological subtype might enable early identification of high-risk patients and could potentially lead to more intensified follow-up or early therapeutic interventions. In particular, tumour grade and AJCC stage can help identify RCC patients at higher risk for rapid progression to pathological fractures, informing individualized monitoring strategies, closer surveillance for skeletal complications, and timely consideration of interventions aimed at reducing fracture risk and preserving function.

The prevalence of fractures in long bones, particularly the humerus, may reflect both anatomical susceptibility and metastatic preference. The rich vascular supply and thinner cortical bone of the humerus may make it especially prone to metastatic involvement. Previous literature has noted differences in survival outcomes between patients with skeletal metastases and those with pathological fractures. In a study by Fottner et al. (2010), for 101 patients who underwent surgical treatment for skeletal metastasis originating from RCC the overall survival rates post-surgery were 58% at 1 year, 37% at 2 years, and 12% at 5 years. The absence of pathological fractures was also shown to be associated with higher survival rates [22].

Our study aimed to identify factors associated with survival both after primary diagnosis and following pathological fracture. Contrary to earlier reports linking age and skeletal location with prognosis, we found no significant influence of fracture location, treatment modality (surgical versus non-surgical), age, or gender on post-fracture survival. These findings underscore the systemic nature of disease progression at this stage and suggest that local treatment decisions should prioritize symptom management and functional preservation rather than survival extension.

Although Fuhrman grade is a recognized prognostic factor following primary diagnosis of RCC, Lin et al. (2007) showed that it does not significantly correlate with survival after surgical treatment for pathological fractures. In their cohort of 295 patients with osseous metastases from RCC, survival was more strongly influenced by factors such as the presence of solitary versus multiple bone metastases and visceral involvement [23]. These findings suggest that once skeletal disease progresses to pathological fracture, tumour grade may have limited prognostic relevance in comparison to disease burden and metastatic pattern.

Although 66% of the patients in our cohort underwent surgical management for their pathological fractures, we observed no significant survival benefit compared to non-surgical treatment. This supports previous literature indicating that surgery in this context serves a primarily palliative role [11]. Nevertheless, surgical intervention may still be appropriate in selected patients with adequate performance status, particularly when aimed at pain relief and restoration of mobility, even in the context of a limited life expectancy. Future studies incorporating treatment variables, functional outcomes, and patient-reported quality of life could provide a more nuanced understanding of the role of surgical intervention in this patient population. Additionally, developing predictive models that incorporate tumour biology, skeletal site, and systemic treatment status may improve risk stratification and aid clinicians in identifying patients who may benefit from early interventions.

## 5 Strengths and limitations

A major strength of this study is the use of two well-established national registries; this ensures a large sample size, high external validity, and detailed clinical information. The combination of fracture-specific and oncological data allowed for a comprehensive analysis of variables that influence both fracture risk and survival.

However, several limitations must be acknowledged. The retrospective design inherently introduces the risk of selection and information bias. Missing data, particularly regarding Fuhrman grade, AJCC stage, and fracture subclassifications, may have influenced the findings. Detailed information on systemic oncological treatments administered before and after pathological fracture, including targeted therapies and immune checkpoint inhibitors, was not available, which may represent a source of residual confounding in survival analyses. Established prognostic risk models, such as the Motzer and IMDC scores, could not be applied due to the lack of clinical and laboratory data, including performance status and haematological parameters. These models are well validated for stratifying outcomes in patients with mRCC [24], but data are limited regarding their predictive performance specifically in patients with bone metastases [12]. Their absence in our cohort limits assessment of whether tumour grade is independently predictive beyond established risk factors.

Furthermore, over the study period, systemic therapy for mRCC has evolved substantially, particularly with immune checkpoint inhibitor–based combinations. Differences in treatment era and therapy type may have influenced both the timing of pathological fractures and post-fracture survival, introducing temporal confounding [25], [26].

Because spinal surgeries in Sweden are traditionally documented in the National Spinal Surgery Register (Swespine) rather than in the Swedish Fracture Register (SFR), there is a substantial risk of underestimating the true number of pathological spinal fractures in the present study. This is particularly relevant given the overlapping clinical and radiological features between pathological vertebral fractures and spinal metastases. In clinical practice, spinal lesions with a suspected or confirmed malignant origin may often be classified and managed primarily as metastatic disease rather than as pathological fractures, and consequently not reported in the SFR. This classification bias could result in an underrepresentation of spinal pathological fractures in SFR, despite them being clinically and therapeutically relevant. Future studies incorporating more detailed data are required to validate these findings.

## 6 Conclusions

Higher Fuhrman grade, advanced AJCC-stage and TNM-stage were associated with a shorter time to pathological fracture in RCC patients. Given the limited survival following fracture, timely risk stratification and individualized management are essential to optimize patient care.

## CRediT authorship contribution statement

**Josefin Åkerstedt:** Writing – original draft, Formal analysis. **Tova Åström:** Writing – review & editing. **David Wennergren:** Writing – review & editing, Methodology. **Johan Wänman:** Writing – review & editing, Supervision, Formal analysis, Conceptualization.

## Declaration of competing interest

The authors declare that they have no known competing financial interests or personal relationships that could have appeared to influence the work reported in this paper.

## Data Availability

The dataset analysed in this study is not publicly available since the study was approved to ensure the confidentiality of patient identifiable data. We are favourable to sharing data but are legally restricted from sharing the data publicly according to the law on Public Access and Secrecy, chapter 21, paragraph 7 and chapter 25, paragraph 1 (https:// www.riksdagen.se/sv/dokument-lagar/dokument/svenskforfattningssamling/offentlighets–och-sekretesslag-2009400_sfs2009–400). Any person interested in the data set may contact Umeå University and the corresponding author to explore ways to share data according to Swedish laws and regulations. It is also possible for individuals interested in this data to apply directly to the Centre of Registers, Västra Götaland (URL: http://registercentrum.se/). This process involves approval from the Swedish Ethical Review Authority.
